# Uncovering the composition of microbial community structure and metagenomics among three gut locations in pigs with distinct fatness

**DOI:** 10.1038/srep27427

**Published:** 2016-06-03

**Authors:** Hui Yang, Xiaochang Huang, Shaoming Fang, Wenshui Xin, Lusheng Huang, Congying Chen

**Affiliations:** 1 State Key Laboratory for Pig Genetic Improvement and Production Technology, Jiangxi Agricultural University, 330045, Nanchang, China

## Abstract

Uncovering the phylogenetic composition of microbial community and the potential functional capacity of microbiome in different gut locations is of great importance to pig production. Here we performed a comparative analysis of gut microbiota and metagenomics among jejunum, ileum and cecum in pigs with distinct fatness. 16S rRNA gene sequencing revealed dramatic differences of microbial composition, diversity and species abundance between small intestine and cecum. *Clostridium* and *SMB53* were enriched in the small intestine, while *Prevotella*, *Treponema*, *Ruminococcus* and *Faecalibacterium* showed a higher abundance in the cecum. Functional capacity analysis of gut microbiome revealed that the microbiome of small intestine plays important roles in the metabolism of small molecule nutrients, while the microbiome of cecum has the stronger ability to degrade xylan, pectin and cellulose. We identified tens of fatness associated-bacterial species including *Escherichia* spp. that showed a notable increase of relative abundance in all three gut locations of high fatness pigs. We further suggested that the potential pathogens, inflammation process, and microbial metabolism and nutrient sensing are involved in the high fatness of pigs. These results improve our knowledge about microbiota compositions in different gut locations, and give an insight into the effect of gut microbiota on porcine fatness.

Mammalian gastrointestinal tract harbors a vast and complex microbial community that plays fundamental roles in many essential processes, such as energy harvest, carbohydrate metabolism and immune development^1^. The microbiome in human gastrointestinal tract is estimated to contain more than 9 million unique genes^2^. This number is approximately 300 times larger than the human gene complement. Therefore, the taxonomic diversity of the microbiota in the gastrointestinal tract is tremendous and may provide numerous biological activities that the host lacks^3^. Recent years, there have been many studies focusing on the investigation of the relationship of gut microbiota with human and mouse obesity. For examples, Backhed *et al.*^4^ first reported that the gut microbiota could modulate lipid metabolism by regulating gene expression. They showed that the suppression of *Fiaf* gene is essential for the microbiota-induced deposition of lipid. Turnbaugh *et al.*^5^ reported that the microbiome of obese mice has an increased capacity to harvest energy by influencing the efficiency of calorie harvest from the diet compared to that of lean mice. Turnbaugh *et al.*^6^ further showed that the human obesity is associated with phylum-level changes in gut microbiota with reduction in bacterial diversity, and 75% of the obesity-enriched genes are from *Actinobacteria* phylum, while 42% of the lean-enriched genes are from *Bacteroidetes*. Fei *et al.*^7^ reported that the endotoxin-producing *Enterobacter* shows the obesity-inducing capacity that may causatively contribute to the development of obesity in its host. Duca *et al.*^8^ indicated that susceptibility to obesity is characterized by an unfavorable microbiome predisposing the host to peripheral and central inflammation, promoting weight gain and adiposity during obesogenic feeding.

Since the similarity of the swine and human in metabolism and physiology^9^, swine has become an important animal model for studying the relationship of gut microbiota with obesity. To date, there have been several studies to investigate the relationship of gut microbiota with fatness in pigs. Wall *et al.*^10^ uncovered that metabolic activity of porcine enteric microbiota affects the fatty acid composition of adipose and liver. Guo *et al.*^11^ showed that obese pigs had a fewer percentage of *Bacteroidetes* division than lean pigs. Further, Guo *et al.*^12^ suggested that body fat is related to the ratio of *Bacteroidetes* division of gut microbiota in the Meishan and Landrace pigs. Luo *et al.*^13^ revealed that lean breed Landrace pigs have a greater diversity and higher numbers of fecal methanogens than obese breed Erhualian pigs. Kim *et al.*^14^ published the longitudinal investigation of pig gastrointestinal microbiome. The study reported by Looft *et al.*^15^ also investigated the microbiota composition and functional capacity of porcine ileum, cecum and mid-colon. But to our knowledge, there are few studies about microbial community structure and functional capacity of microbiome in other sections of small intestine of pigs, such as jejunum. Gut microbiota involves in energy harvest and carbohydrate metabolism^1^. Recent studies have shown that gut microbiota has an important impact on the efficiency of nutrient uptake and energy utilization^16^. We suggest that gut microbial community structure and metagenome have essential effects on porcine production traits, such as fat deposition. However, there have been few studies about that. It is of great importance to uncover the distinct microbiota and functional capacity of microbiome related to fat deposition in pigs.

In this study, we aimed to use 16S rRNA gene sequencing and high-throughput metagenomic sequencing approach to elucidate the microbial community structure and potential functional capacity of metagenomics in the jejunum, ileum and cecum in a Chinese indigenous pig breed of Laiwu, which exhibits the characteristics of the strong ability of fat deposition. We compared the microbial composition and potential functional capacity of microbiomes among three gut locations and evaluated the effect of gut microbiota on porcine fat deposition.

## Results

### Significant difference of phylogenetic compositions among three gut locations of pigs

**Figure 1:**
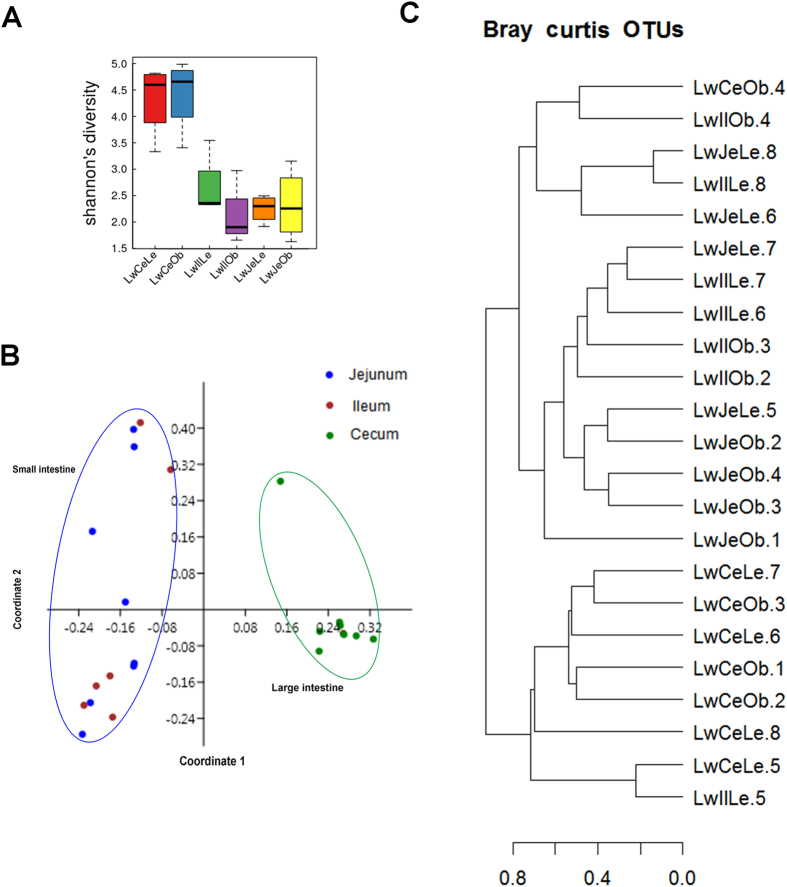
Alpha- and beta-diversity comparison of the microbiomes of the jejunum, ileum and cecum. Analyses were performed on 16S rRNA V4 region data. Sample names are coded by breed (Lw, Laiwu breed), sampling site (Je, Jejunum; Il, Ileum; Ce, Cecum) and fat deposition status (Ob, high fatness; Le, low fatness). For example, LwIlOb represented the sample that was collected from the ileum of Laiwu pig with high fatness. The samples with the same Arabic numeral in the sample name were harvested from the same pig. (**A**) Alpha-diversity comparison based on shannon’s diversity index, grouped by sampling site and fat deposition status (mean ± SEM). Compared to the ileum and jejunum, the cecum has the significantly higher microbial richness. (**B**) Unweighted UniFrac principal coordinate analysis by bacterial microbiota. The small intestine (ileum and jejunum) and cecum samples show clear separation (**C**) Bray-curtis cluster tree. The samples from the same gut location trend to cluster together.

To characterize the phylogenetic composition of bacterial communities in different gut locations, we firstly performed the 16S rRNA gene sequencing analysis and compared the alpha-diversity of microbiota among jejunum, ileum and cecum. The cecum had a significantly higher Shannon index (taking into account the number of species and the evenness of the species) than the jejunum and ileum (*P* < 0.05, Fig. 1A). However, the similar alpha-diversity was observed between jejunum and ileum.

We then explored the variation of phylogenetic diversity among all tested samples. Except the sample LwIlLe.5 that was clustered into the samples from the cecum, all other samples showed distinct clustering between small intestine (jejunum and ileum) and cecum in unweighted principal coordinate analysis (PCoA) (Fig. 1B). Similarly, although the phylogenetic composition of the LwCeOb.4 from the cecum was similar to that of the samples from the ileum, and the sample LwIlLe.5 from the ileum showed similarity with the cecum samples, most of samples showed distinct phylogenetic composition between small intestine and cecum in Bray Curtis cluster analysis (Fig. 1C). Different beta-diversity of the above two samples should be due to the states of fatness (see below). However, we did not observe the distinct clustering between samples from the jejunum and ileum.

**Figure 2:**
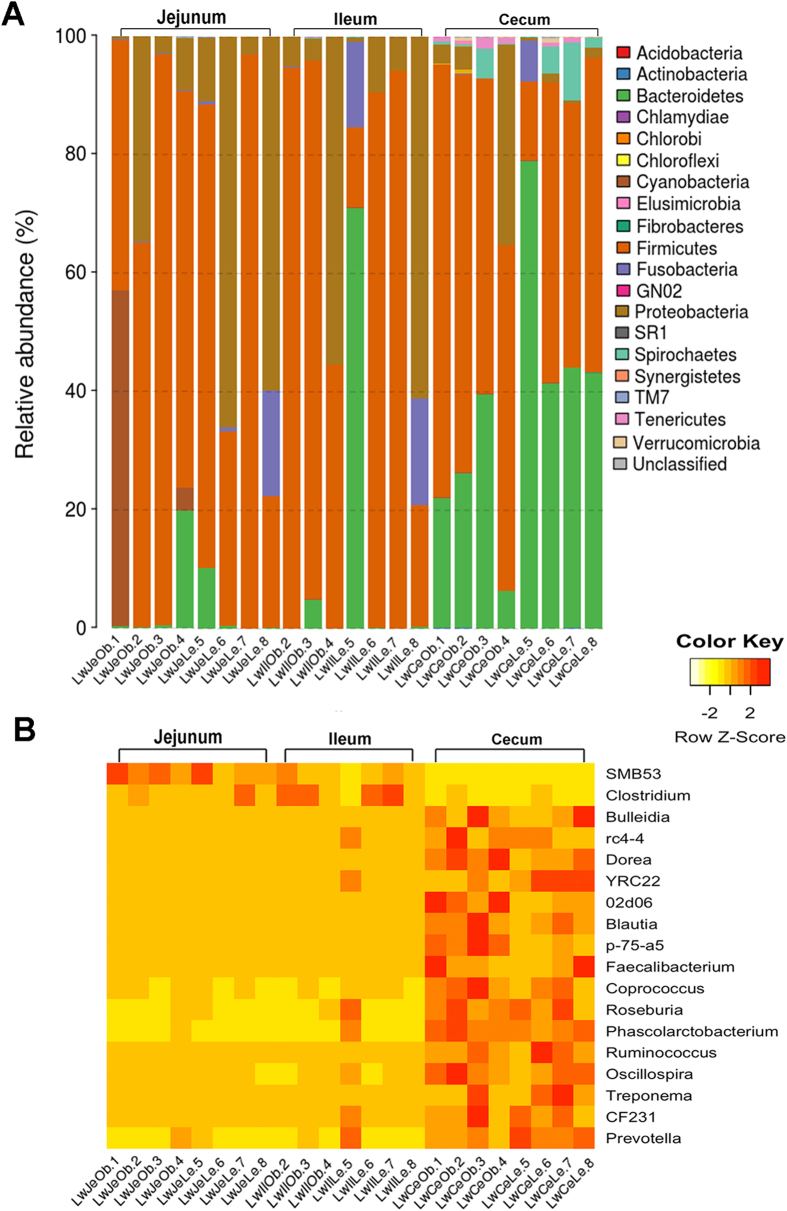
Taxonomic profiles of the microbial communities of the jejunum, ileum and cecum derived from 16S rRNA gene sequencing data. (**A**) Microbial composition at the phylum level. Samples are represented along the horizontal axis, and relative abundance is denoted by the vertical axis. (**B**) Heatmap showing the 18 genera with significant differences of relative abundances among three gut locations. Heatmap is color-coded based on row z-scores.

Furthermore, we focused on the taxonomic distribution of the numerically abundant bacteria derived from the 16S rRNA gene sequences in each gut location. At the phylum level, *Firmicutes* was the highest enriched phylotype in the jejunum and ileum, which accounted for 60.0% and 65.8% of relative abundance, respectively, followed by *Proteobacteria*, which occupied 29.4% and 20.3% of abundance (Fig. 2A). However, in the cecum, *Firmicutes* and *Bacteroidetes* were the two predominant phyla, which showed 51.7% and 37.6% of relative abundance, respectively. In addition, we also noticed *Spirochaetes*, which occupied 2.7% of microbiota composition in the cecum and had a significantly higher relative abundance than that in the small intestine (Fig. 2A). At the genus level, *Clostridium, SMB53* and *Escherichia* were the top three genera in the small intestine, while *Prevotella*, *Escherichia* and *Lactobacillus* were the most abundant genera in the cecum (Supplementary Fig. S1). We further performed Kruskal-Wallis test to compare the relative abundance of bacterial genera among three gut locations. We identified 18 genera showing significant differences of relative abundances, including two genera having different abundance among three locations and 16 genera between small intestine and cecum (Fig. 2B and Supplementary Table S1). Only *Clostridium* and *SMB53* were significantly enriched in the jejunum and ileum (*P* < 0.05). *Clostridium* showed a 14.66% and 27.14% of relative abundance in the jejunum and ileum, respectively. And the *SMB53* had 36.70% and 14.54% of relative abundance, while none of them showed the relative abundance above 3.0% in the cecum (Fig. 2B and Supplementary Table S1). The other 16 genera were significantly enriched in the cecum, including *Prevotella*, *Ruminococcus*, *Treponema* and *Blautia.*

Metagenomic sequencing data was also used to compare the microbial community structure among jejunum, ileum and cecum. Total 6 luminal DNA pools from three gut locations of Laiwu pigs were used to construct libraries for metagenomic sequencing. Each location provided two DNA pools including one pool from pigs with high fatness and the other from pigs with low fatness (methods). We obtained an average of 11.43 Gb of raw data for each DNA pool, ranging from 9.22 Gb to 14.29 Gb (Table 1). After filtering the low quality reads and the host pollution, we obtained 3.53 Gb to 5.01 Gb of clean data for each sample. We performed *de novo* assembly for all short sequence data using SOAPdenovo assembler (Version 1.06)^17^. The contig numbers of six DNA pool samples ranged from 40,420 to 139,978 with the contig length >500 bp (Table 1). MetaGeneMark (MGM) program was used to predict open reading frame (ORF) of each contig (see Methods). The six samples contained a total of 1,056,020 ORFs that were longer than 100 bp (Table 1).

**Figure 3:**
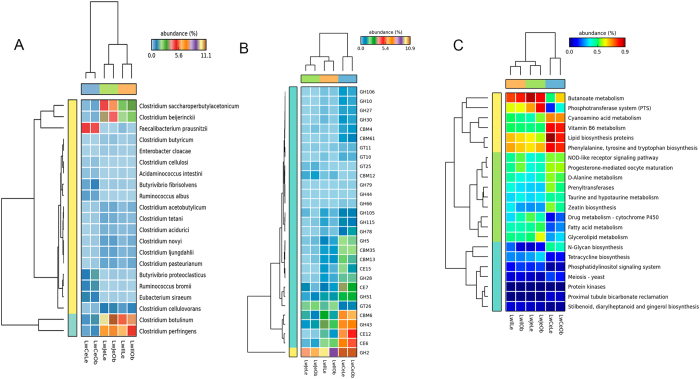
Heatmap of gut luminal samples for bacterial species, CAZy function terms and KEGG subsystems from metagenomic sequencing data. (**A**) Heatmap depicts the relative abundances of the 21 bacterial species significantly enriched in jejunum, ileum and cecum. (**B**) Heatmap of CAZy functional terms that showed significantly different enrichments among jejunum, ileum and cecum. GH: Glycoside Hydrolase, GT: GlycosylTransferase, PL: Polysaccharide Lyase, CE: carbohydrate esterases, CBM: Carbohydrate-Binding Module. (**C**) Heatmap of KEGG subsystems significantly enriched in jejunum, ileum and cecum.

We then compared the microbial community structure among three gut locations based on metagenomic sequencing data. The microbiota of all three gut locations was dominated by bacteria kingdom, which occupied more than 90% of total short read sequences. The microbial composition was quite similar to that in 16S rRNA gene sequencing analysis at the phylum level (Supplementary Fig. S2). Metagenomic sequencing analysis improved the phylotype resolution from genus level to the species level. At the significant threshold of *P* < 0.05 and *q* < 0.2, we identified a total of 21 bacterial species showing different enrichments among three gut locations. Among them, eight species were only enriched in the cecum, five species showed the higher abundance in the jejunum and ileum, and the other eight species were successively reduced their relative abundances from anterior to posterior (Fig. 3A and Supplementary Table S2). We further observed that 12 out of the 13 species that had higher abundances in the jejunum and ileum belong to *Clostridium,* while the bacterial species enriched in the cecum have been reported to associate with fibre fermentation and butyrate-producing, such as *Ruminococcus spp*.^18^, *Faecalibacterium prausnitzii*^19^ and *Butyrivibrio fibrisolvens*^20^ (Fig. 3A and Supplementary Table S2).

### Comparison of functional capacity of gut microbiome between small intestine and cecum

To compare the potential functional capacity of microbiome among three gut locations, we blasted the predicted ORFs against CAZy^21^ and KEGG^22^ database. Overall, about 1.1% ~ 2.5% of genes could be classified into CAZy database, and about 25.8% ~ 41.6% of genes were assigned to KEGG pathways. ANOVA test was then used to compare the potential functional capacity of gut microbiome among three intestinal locations. We identified a total of 29 CAZy families that showed significantly different enrichments in different intestinal locations (*P* < 0.05, Fig. 3B). Twenty-one out of these 29 CAZy families were significantly enriched in the cecum (Supplementary Table S3), including esterase families CE6 (acetyl xylan esterase), CE7 (acetyl xylan esterase) and CE12 (pectin acetylesterase) that are associated with xylan, pectin and hemicellulose degradation. GH43, GH51 and GH28 that belong to glycoside hydrolases families were showed successive increase of relative abundance from anterior to posterior. However, GT25, GT26 and CBM12 which play important roles in catalyzing utilization of monosaccharide and assembly of glycoconjugates and complex carbohydartes^23^ were significantly enriched in the small intestine. Furthermore, we identified 22 KEGG pathways that showed different enrichments in different intestinal locations (Fig. 3C and Supplementary Table S4). Eight out of these 22 KEGG pathways were significantly enriched in the small intestine, including butanoate metatbolism, phosphotransferase system (PTS), fatty acid metabolism, phosphatidylinositol signaling system, glycerolipid metabolism and tetracycline biosynthesis (Supplementary Table S4). Most of genes that were assigned to the above functional categories were derived from the genus *Clostridium*. For example, 47.6% and 44.9% genes assigned to PTS pathway were derived from *Clostridium* in the jejunum and ileum, respectively. However, cyanoamino acid metabolism, vitamin B6 metabolism, lipid biosynthesis proteins, N-Glycan biosynthesis and the other 10 subsystems were significantly enriched in the cecum (Supplementary Table S4).

### Comparison of microbial community structure between pigs with distinct fatness in different intestinal locations

Because the gut luminal samples for 16S rRNA gene and metagenomic sequencing were collected from pigs with distinct backfat thickness, we could evaluate the microbial species potentially related to porcine fat deposition. We firstly performed PCA analysis at the phylum level with 16S rRNA gene sequencing data. Only the samples from the cecum showed significant differences of microbial structures between high and low fatness pigs (Supplementary Fig. S3). We further compared the relative abundance of each microbe between high and low fatness pigs. Compared to the high fatness pigs, the pigs with low fatness had a significantly higher abundance of Bacteroidetes, but a lower abundance of *Firmicutes* in the cecum (*P* < 0.05). Although we did not identify any bacterial phylum that showed significantly different abundance between high and low fatness pigs in the small intestine, *Bacteroidetes* and *Firmicutes* showed a tendency that had different abundance between high and low fatness pigs (Supplementary Fig. S2). At the genus level, we only identified *YRC22* (0.61 ± 0.22 *vs.* 0.10 ± 0.12), *Prevotella* (28.87 ± 11.83 *vs.* 9.22 ± 3.05) and *Paludibacter* (0.61 ± 0.37 *vs.* 0.05 ± 0.04) that showed the higher abundances in the cecum of the low fatness pigs (*P* < 0.05, *q* < 0.2), while none such microbes were identified in the jejunum and ileum.

Subsequently, based on the metagenomic sequencing data, we identified 8, 19 and 15 bacterial species that showed significant differences of relative abundances between high and low fatness pigs in the jejunum, ileum and cecum, respectively (Supplementary Table S5). In the jejunum, *Clostridium phytofermentans, Lactobacillus johnsonii* and *Escherichia fergusonii* were enriched in the pigs with high fatness, while succinic-producing bacteria (*Actinobacillus succinogenes* and *Mannheimia succiniciproducens*) and butyrate-producing bacteria (*Cellulosilyticum lentocellum*)^24^ had more abundance in the low fatness pigs. In the ileum, high fatness pigs had the significant enrichments for the opportunistic pathogens of *Escherichia coli* (16.96 *vs.* 4.64), *Clostridium difficile* (16.02 *vs.* 6.07) and *Clostridium sordellii* (15.29 *vs.* 5.68). However, three *Prevotella* species, seven *Bacteroides* species, *Actinobacillus succinogenes* and *Cellulosilyticum lentocellum* were significantly enriched in the low fatness pigs. In the cecum, *Escherichia coli* (2.76 *vs.* 0.72)*, Roseburia hominis* (1.75 *vs.* 0.73), *Roseburia intestinalis* (3.22 *vs.* 1.24) and *Oscillibacter valericigenes* (6.62 *vs.* 1.38) were significantly enriched in high fatness pigs, while the low fatness pigs were enriched for *Prevotella ruminicola*, *Treponema succinifaciens* and five *Bacteroides* species (Supplementary Table S5).

### Comparative functional capacity of gut microbiomes between high and low fatness pigs

**Figure 4:**
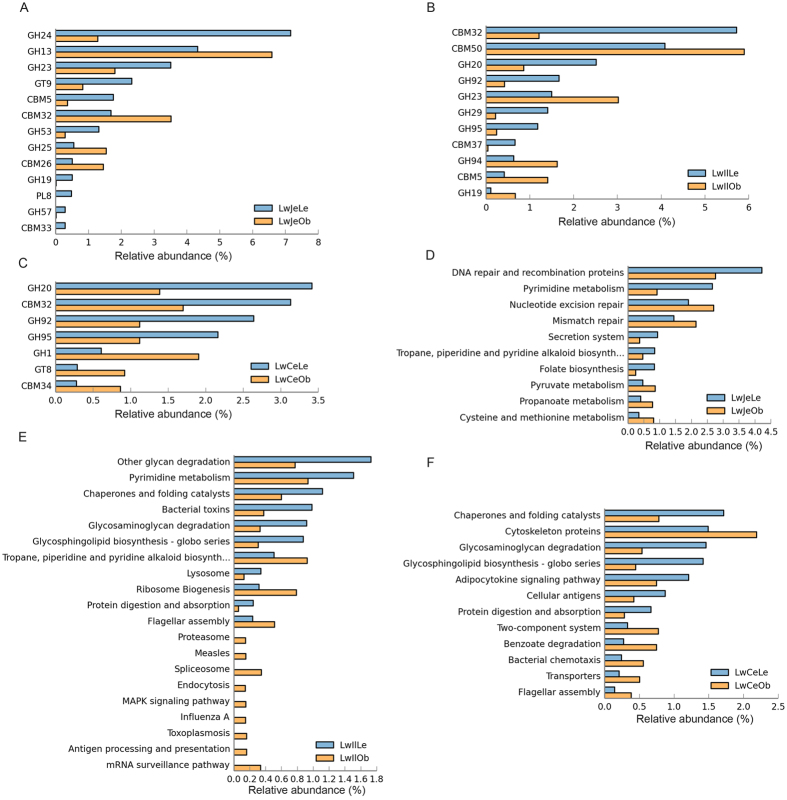
Comparison of functional capacities of the gut microbiomes between high and low fatness pigs. (**A**–**C**) show the comparisons of the relative abundances of CAZy functional terms between high and low fatness pigs in jejunum, ileum and cecum, respectively. GH: Glycoside Hydrolase, GT: GlycosylTransferase, PL: Polysaccharide Lyase, CE: carbohydrate esterases, CBM: Carbohydrate-Binding Module. (**D**–**F**) indicate the comparisons of the relative abundances of KEGG subsystems between high and low fatness pigs in jejunum, ileum and cecum.

We then compared the functional capacity of gut microbiome between high and low fatness pigs through functional annotation of metagenome with the CAZy and KEGG database. In the jejunum, a total of 13 CAZy subsystems showed significant difference of relative abundance between low and high fatness pigs. The high fatness pigs had significant enrichments for the subsystems related to starch and lactose degradation, such as GH13, CBM26 and CBM32 (Fig. 4A), while the low fatness pigs were significantly enriched for nine CAZy subsystems including GH24 and GH23 related to lysozyme, and GH19 and CBM5 that are associated with chitinase degradation. In the ileum, five CAZy subsystems were significantly enriched in the high fatness pigs, including CBM50, GH23, GH94, CBM5 and GH19 that are related to the cleaving of chitin and peptidoglycan, while CBM32, CBM37, GH20, GH29, GH92 and GH95 showed higher relative abundances in the low fatness pigs (Fig. 4B). In the cecum, seven CAZy subsystems showed significant differences of relative abundances between high and low fatness pigs. The subsystems significantly enriched in the low fatness pigs were similar to that in the ileum (including CBM32, GH20, GH92 and GH95; Fig. 4C), while the high fatness pigs had significant enrichments of GH1, CMB34 and GT8 (involved in lipopolysaccharide biosynthesis^25^).

KEGG annotation was used to further compare the functional capacity of gut microbiome between low and high fatness pigs. In the jejunum, genes related to pyruvate metabolism, propanoate metabolism, and cysteine and methionine metabolism were significantly enriched in the metagenome of high fatness pigs (Fig. 4D). However, genes associated with pyrimidine metabolism, secretion system and folate biosynthesis had more abundance in the low fatness pigs. In the ileum, genes related to glycan degradation, pyrimidine metabolism, glycosaminoglycan degradation and glycosphingolipid biosynthesis had the higher abundance in the low fatness pigs (Fig. 4E), while antigen processing and presentation, influenza A, MAPK signaling pathway and endocrtosis were significantly enriched in the high fatness pigs. In the cecum, adipocytokine signaling pathway, cellular antigen, and protein digestion and absorption showed higher enrichments in the low fatness pigs. However, two-component system, benzoate degradation and bacterial chemotaxis were significantly enriched in the metagenomes of high fatness pigs (Fig. 4F).

## Discussion

There have been several studies about the gut microbial composition of pigs based on 16S rRNA gene sequencing^26^,^27^,^28^. To our knowledge, there are few reports about pig metagenomic sequencing analysis that can evaluate the microbial community structure at species level in different gut locations. In this study, we compared microbial community structure in three gut locations, characterized the functional capacity of the swine microbiome from anterior to posterior, and investigated the potential relationship of gut microbiome with porcine fatness by high-throughput next generation sequencing. To our knowledge, this is the first report about the comprehensive analysis of microbial community structure of porcine jejunum and the evaluation of gut bacterial species related to porcine fatness.

Each gut location is functionally and anatomically diverse. Compositions of microbial community structure throughout the intestinal system should be varied by location. At the phylum level, Isaacson *et al.*^29^ identified that *Firmicutes* represents more than 95% of the bacteria detected in the ileum, and *Firmicutes* and *Bacteroidetes* occupy greater than 90% of the bacteria detected in the cecum. In this study, *Firmicutes* was the highest enriched phylotype in the ileum, but it only accounted for 65.8% of relative abundance. *Firmicutes* and *Bacteroidetes* were the two predominant phyla in the cecum, which showed 51.7% and 37.6% of relative abundance. Looft *et al.*^15^ found that differences of bacterial compositions between the ileum and colon were the results of the dominant genera *Anaerobacter* and *Turicibacter* in the ileum, and *Prevotella*, *Oscillibacter* and *Succinivibrio* in the colon. However, in this study, *Clostridium* was the top genus in the ileum and showed significantly different abundance between the ileum and cecum, while *Prevotella* was most abundant and significantly enriched in the cecum (Supplementary Fig. S1). This discrepancy may be due to different breed, age (90 days *vs.* 300 days) and environment factors. We observed the similar microbial community structure between swine jejunum and ileum. Mao *et al.*^30^ also indicated a similar microbial composition between bovine jejunum and ileum. However, similar to the previous reports in human and swine^31^,^32^, the microbiomes of jejunum and ileum are less complex than that of cecum (Fig. 1). The numbers of functional genes annotated to KEGG and CAZy subsystems in the metagenomes of jejunum and ileum were also significantly lower than that in cecum.

In the KEGG analysis, phosphotransferase system (PTS) was significantly enriched in the small intestine. PTS is a complex group translocation system presenting in many bacteria. It transports sugars (such as mannose, glucose and mannitol) into the cell and phosphorylates the substrate via phosphotransferase during transport. Corresponding to this result, CAZy functional annotations of the metagenomes also revealed that the microbiome of small intestine tends to metabolize small molecule nutrients, such as monosaccharide. Zoetendal *et al.*^32^ also suggested that rapid uptake and fermentation of simple carbohydrates (such as glucose and maltose) contribute to maintaining of the microbiota in the small intestine. Most of genes that were assigned to PTS pathway were derived from *Clostridium* in the small intestine. This result was consistent with that 12 species of *Clostridium* were enriched in the jejunum and ileum. It has been known that many Clostridia can digest whey and sugar yielding butanol, propionic acid, ether, and glycerin. Actually, the subsystems of butanoate metabolism and fatty acid metabolism had the higher abundances in the jejunum and ileum (Fig. 3C).

Until now, there are few studies about the effects of gut microbiota on swine production traits. We investigated the potential relationship of porcine gut microbiome with fat deposition. Just like in humans^33^,^34^,^35^, the high fatness pigs showed a lower abundance of Bacteroidetes and a higher abundance of Firmicutes than pigs with low fatness in the large intestine. *Escherichia* spp. was identified to significantly enrich in the high fatness pigs in all three gut locations (Supplementary Table S5). The study in the mouse model has shown that lipopolysaccharide endotoxin from *Escherichia* coli could induce obese and insulin-resistant phenotypes^36^. The high fatness pigs had the higher abundance of *Clostridium* spp. in both jejunum and ileum. *Clostridium difficile* and *Clostridium* sordellii could lead to infection and inflammation^37^ that is associated with progression of obesity^38^. The previous study also reported that *Clostridium* was significantly increased in the obese mice fed high-fat diet^39^. Interestingly, in the KEGG analysis, several subsystems related to inflammation process, such as MAPK signaling pathway, endocytosis and antigen processing were significantly enriched in the ileum of high fatness pigs (Fig. 4E). These findings suggested that the potential pathogens (e.g. *Escherichia* spp.) and the inflammation process are involved in the high fatness of pigs. Many studies in humans and mice have also indicated that obesity is associated with inflammation^40^,^41^. Bacterial interactions promote intestinal inflammation which correlates with obesity and insulin resistance in high-fat dieted mice^38^.

The high fatness pigs had the significantly lower abundance of *Prevotella* spp. and *Bacteroides* spp. in both ileum and cecum. Ivarsson *et al.*^42^ reported that the abundance of *Bacteroides*–*Prevotella*–*Porphyromonas* in growing pigs was positively correlated with the capacity of fermenting polysaccharides to short-chain fatty acids (SCFAs). The study in mice transplanting human fecal microbiota from twins discordant for obesity revealed that increasing intestinal SCFAs by the lean co-twin’s microbiota influenced host energy balance, inhibited the accumulation of fat in adipose tissue and promoted leptin level^43^. Furthermore, *Bacteroides*-*Prevotella* showed a negative correlation with fat mass development and inflammation in diet-induced obese mice^44^.

On the other hand, microbial metabolism and nutrient sensing (metabolic reaction) should be also related to pig fatness. In the jejunum, several metabolism-related subsystems e.g. cysteine and methionine metabolism, were enriched in the high fatness pigs. In the cecum, the KEGG pathways that link to cell motility (flagellar assembly) and membrane transport (two-component system and transporters) were enriched in the high fatness pigs. These KEGG categories were also reported to be mainly enriched in the distal colon microbiome of obesity mouse and human^5^,^8^,^45^, and the high-fat diet induced murine^39^. The two-component system is involved in signal transduction mechanism and bacterial chemotaxis^46^,^47^, and may be as a metabolic reaction center coupling nutrient sensing to dynamic regulation of monosaccharide metabolism^48^.

In conclusion, we observed distinct microbial community structure between small intestine and cecum, and identified tens of bacterial species that showed significant differences of relative abundances in each gut location. We found the distinct functional capacity of microbiome between small intestine and cecum. We suggested that the potential pathogens (e.g. *Escherichia* spp.), inflammation process, and microbial metabolism and nutrient sensing are involved in the high fatness of pigs. Furthermore, the higher abundances of *Bacteroides* and *Prevotella* may inhibit the fat mass development and inflammation in the low fatness pigs. These results may provide an interesting insight into the complexity of swine gastrointestinal microbial community and help us better understand the effects of gut microbiota on pig production traits.

## Materials and Methods

### Animals and gut luminal sample collection

A total of 330 Laiwu pigs were raised in a fattening house which was comprised of 50 pens, and each pen housed 6 ~ 8 pigs. Pigs were fed two times a day using the corn-soybean feed including 16% of crude protein, 3100 kJ of digestible energy and 0.78% of lysine. Water was available *ad libitum* from nipple drinkers. In addition, swine were supplemented with feed antibiotics (chlortetracycline 50 g per ton, salinomycin 50 g per ton) for improving growth performance. All animals were healthy and did not receive any antibiotic treatment within 2 months before slaughter. All pigs were slaughtered at 300 ± 3 days after fasting and water-free overnight. Eight pigs, which comprised 4 individuals (2 male and 2 female) with the highest backfat thickness (4.25 ± 0.32 cm) and 4 animals (2 male and 2 female) with the lowest backfat thickness (2.52 ± 0.08 cm), were selected for this study. The eight experimental pigs were from different pens and collected the luminal samples at the same site of each gut location. In brief, the entire digestive tract was peeled from the enterocoelia. The luminal contents of jejunum and ileum were separately gathered from the middle part of jejunum and ileum. Cecum luminal samples were collected from the bottom of the cecum. All samples were harvested within 30 min after slaughter. After dipped in liquid nitrogen, the luminal samples were transferred into −80 °C refrigerator until use. All animal work was conducted according to the guidelines for the care and use of experimental animals established by the Ministry of Agriculture of China. The project was also approved by Animal Care and Use Committee (ACUC) in Jiangxi Agricultural University.

### Luminal DNA extraction and 16S rRNA gene sequencing analysis

The luminal DNA was extracted using the QIAamp Fast DNA Stool Mini Kit (Qiagen, Germany) according to the manufacturer’s instructions. The DNA concentration and its quality were measured by Nanodrop-1000 and agarose gel electrophoresis. The fusion primers 515F (5′-GTGCCAGCMGCCGCGGTAA) and 806R (5′-GGACTACHVGGGTWTCTAAT) with dual index were used for amplifying the V4 region of bacterial 16S rRNA gene under the melting temperature of 56 °C with 30 cycles. The sequencing was performed on the Illumina MiSeq platform (Illumina, USA). Because of unsuccessful sequencing for one ileal sample from high backfat thickness pig, the sequencing data from 23 samples were used for further analyses. Paired-end reads were assembled into tags using FLASH (v.1.2.11)^49^. Tags were clustered into operational taxonomic units (OTUs) at 97% similarity using USEARCH software (v7.0.1090)^50^. Taxonomic assignments for the 16S rRNA gene sequences were performed by using the RDP classifer program (v2.2)^51^. Shannon diversity index, Bray-Curtis distant coefficient and unweighted UniFrac were all calculated using QIIME^52^. Kruskal-Wallis test^53^ was used to compare the microbial composition among three intestinal locations. Z-scores were calculated to construct heatmap for showing the relative abundance of the microbes in each sample with the formula Z = (*x* − μ)/σ, where *x* is the relative abundance of microbes in each sample, μ is the mean value of relative abundances of microbes in all samples, and σ is the standard deviation of relative abundances. The comparison of relative abundances of microbes between high and low fatness pigs was performed by Welch’s t-test in STAMP software^54^. Tukey-Kramer post-hoc test was used for pairwise comparisons to find out which group differs. Benjamini-Hochberg FDR method (q value) was used to correct the multiple comparisons^55^. The effected size of each microbe (η^2^) was calculated in the multiple group comparison analysis with STAMP software, the calculation formula was η^2^ = **SS**_between treatments_/(**SS**_between treatments_ + **S**S_within treatments_), where **SS**_between treatments_ is the sum of squares among treatments and **SS**_within treatments_ is the sum of squares within treatment. All 16S rRNA gene sequencing data were deposited in NCBI’s Short Read Archive. The accession numbers are shown in Supplementary Table S6.

### Construction of DNA pools and libraries for metagenomic sequencing

Two DNA pools for each of the jejunum, ileum and cecum were constructed with the same amount of luminal DNA from each animal and provided for metagenomic sequencing, including one DNA pool from 4 high fatness pigs and the other DNA pool from 4 low fatness pigs. DNA libraries for metagenomic sequencing were constructed following the manufacturer’s instruction (Illumina, USA). Each DNA pool was constructed a paired-end library with insert size of ~350 base pairs. We used the same protocol as described by Qin *et al.*^56^ to perform cluster generation and sequencing on a Hiseq 2000 platform (Illumina, USA). The metagenomic sequencing data were deposited in NCBI’s Short Read Archive (Accession numbers in the Supplementary Table S6).

### *De novo* assembly of short reads

We firstly processed the raw data to filter the low quality and host polluted sequences based on the internal procedure: I. The reads containing more than three unknown bases or continuous low-quality bases were removed; II. The adapter and duplication pollutions were trimmed; III. The reads mapped to pig reference genome sequence were removed from further analysis. And then, *de novo* assembly of the high-quality short reads was performed by SOAP *de novo* assembler (v1.06)^17^. The continuous sequences with unambiguous connections were considered as contigs.

### Gene prediction and functional classification

MetaGeneMark (MGM, v2.10) was applied to predict the whole range of ORFs from the contigs of each sample^57^. We used cd-hit software (v4.6.1) to exclude the redundant genes from all the predicted ORFs^58^. A pair of genes with aligned length covering greater than 90% of shorter gene and >95% identity were grouped together. And then, the group sharing genes were merged. The longest gene represented the merged group. The other members of the group were set as redundancy. The non-redundant gene set was constructed by removing the redundancy. Finally, the non-redundant genes were aligned to the protein sequences of KEGG^22^ (v59) and CAZy^21^ (carbohydrate-active enzymes) database using BLAST tool (v2.2.21) with e-value ≤ 1 × 10^−5^ and an identity threshold value ≥40%. The annotated genes were assigned into KEGG pathways. To trace back the genes to microbes, the protein sequences of genes were aligned to the released bacterial reference protein database using the blastp tool with e-value ≤ 1 × 10^−40^. The alignment with the highest score was kept for the further analyses.

### Phylogenetic composition analysis based on metagenomic sequencing data

A non-redundant reference gene catalogue of pig microbiota was constructed through downloading the known bacteria, fungi, viruses and archaebacteria sequences. SOAP aligner (v2.21) was used to map the high-quality read sequences into the microbial communities against the reference gene catalogue^59^. According to the central limit theorem, the mean values of microbial relative abundances in the tested samples that were sampled from a population obey normal distribution. ANOVA test was used to compare the phylogenetic composition among three gut locations. Fisher’s exact test was used for two sample comparison between high and low fatness pigs. The microbes with the ratio of relative abundance <2 and the difference of relative abundance <1 between two groups were filtered from differential microbes. Tukey-Kramer *post-hoc* test was used for pairwise comparisons. Story’s FDR was used to correct multiple comparisons^60^. All tests were performed using STAMP software^53^.

### Comparison of functional capacity of gut microbiomes

To compare the functional capacity of gut microbiomes, the abundance of the annotated genes was normalized as follows: (the count of reads mapped to each gene/total number of clean reads)*100000. The relative abundance of each subclass term of the CAZy families and KEGG pathways was calculated by summing the abundance of these genes that were annotated to the functional subsystem. The comparisons of functional capacity profiles between high and low fatness pigs were performed by Fisher’s exact test. ANOVA test was used to compare the functional capacity of microbiomes among three gut locations. Z-scores were calculated to construct the heatmaps for showing the relative abundances of KEGG and CAZy subsystems in each sample. Tukey-Kramer post-hoc test was used for pairwise comparisons. Story’s FDR was used to correct multiple comparisons^60^. All the tests were performed by using STAMP software^53^.

## Additional Information

**How to cite this article**: Yang, H. *et al.* Uncovering the composition of microbial community structure and metagenomics among three gut locations in pigs with distinct fatness. *Sci. Rep.* **6**, 27427; doi: 10.1038/srep27427 (2016).

## Supplementary Material

Supplementary Information

## Figures and Tables

**Table 1:** Summary of metagenomic sequencing data obtained in this study.

ID	Raw data (Mbp)	Clean data (Mbp)	Contig number	N50 (bp)	ORF Number	Average length of ORF (bp)	Percentage of ORFs mapped to KEGG	Percentage of ORFs mapped to CAZy
LwJeOb	10,177	3,527	57.148	2,264	128,294	625.08	39.85%	1.90%
LwJeLe	12,849	4,115	46,886	2,031	101,423	620.95	39.59%	1.69%
LwIlOb	9,228	3,573	40,420	5,845	99,935	531.24	25.80%	1.11%
LwIlLe	10,981	4,686	103,560	2,226	222,880	659.90	38.48%	2.23%
LwCeOb	11,058	4,907	139,978	1,335	262,320	559.17	33.74%	1.46%
LwCeLe	14,289	5,011	120,389	1,849	241,168	643.84	41.64%	2.46%

## References

[b1] Greenblum S., Turnbaugh P. J. & Borenstein E. Metagenomic systems biology of the human gut microbiome reveals topological shifts associated with obesity and inflammatory bowel disease. Proc Natl Acad Sci USA 109, 594–599 (2012). 22184244 10.1073/pnas.1116053109 PMC3258644

[b2] Yang X., Xie L., Li Y. & Wei C. More than 9,000,000 unique genes in human gut bacterial community: estimating gene numbers inside a human body. PLoS One 4, e6074 (2009). 19562079 10.1371/journal.pone.0006074 PMC2699651

[b3] Backhed F., Ley R. E., Sonnenburg J. L., Peterson D. A. & Gordon J. I. Host-bacterial mutualism in the human intestine. Science 307, 1915–1920 (2005). 15790844 10.1126/science.1104816

[b4] Bäckhed F. *et al.* The gut microbiota as an environmental factor that regulates fat storage. Proc Natl Acad Sci USA 101, 15718–15723 (2004). 15505215 10.1073/pnas.0407076101 PMC524219

[b5] Turnbaugh P. J. *et al.* An obesity-associated gut microbiome with increased capacity for energy harvest. Nature 444, 1027–31 (2006). 17183312 10.1038/nature05414

[b6] Turnbaugh P. J. *et al.* A core gut microbiome in obese and lean twins. Nature 457, 480–484 (2009). 19043404 10.1038/nature07540 PMC2677729

[b7] Fei N. & Zhao L. An opportunistic pathogen isolated from the gut of an obese human causes obesity in germfree mice. ISME J 7, 880–884 (2013). 23235292 10.1038/ismej.2012.153 PMC3603399

[b8] Duca F. A. *et al.* Replication of obesity and associated signaling pathways through transfer of microbiota from obese-prone rats. Diabetes 63, 1624–1636 (2014). 24430437 10.2337/db13-1526

[b9] Chen C. *et al.* A global view of porcine transcriptome in three tissues from a full-sib pair with extreme phenotypes in growth and fat deposition by paired-end RNA sequencing. BMC Genomics 12, 448 (2011). 21906321 10.1186/1471-2164-12-448 PMC3188532

[b10] Wall R. *et al.* Metabolic activity of the enteric microbiota influences the fatty acid composition of murine and porcine liver and adipose tissues. Am J Clin Nutr 89, 1393–1401 (2009). 19357220 10.3945/ajcn.2008.27023

[b11] Guo X. *et al.* Development of a real‐time PCR method for Firmicutes and Bacteroidetes in faeces and its application to quantify intestinal population of obese and lean pigs. Lett Appl Microbiol 47, 367–373 (2008). 19146523 10.1111/j.1472-765X.2008.02408.x

[b12] Guo X., Xia X., Tang R. & Wang K. Real-time PCR quantification of the predominant bacterial divisions in the distal gut of Meishan and Landrace pigs. Anaerobe 14, 224–228 (2008). 18524640 10.1016/j.anaerobe.2008.04.001

[b13] Luo Y.-h. *et al.* Lean breed Landrace pigs harbor fecal methanogens at higher diversity and density than obese breed Erhualian pigs. Archaea 2012, 489–506 (2012). 10.1155/2012/605289 PMC3403511 22844227

[b14] Kim H. B. *et al.* Longitudinal investigation of the age-related bacterial diversity in the feces of commercial pigs. Vet Microbiol 153, 124–133 (2011). 21658864 10.1016/j.vetmic.2011.05.021

[b15] Looft T. *et al.* Bacteria, phages and pigs: the effects of in-feed antibiotics on the microbiome at different gut locations. ISME J 8, 1566–1576 (2014). 24522263 10.1038/ismej.2014.12 PMC4817603

[b16] Delzenne N. M. & Cani P. D. Interaction between obesity and the gut microbiota: relevance in nutrition. Annu Rev Nutr 31, 15–31 (2011). 21568707 10.1146/annurev-nutr-072610-145146

[b17] Li R. *et al.* *De novo* assembly of human genomes with massively parallel short read sequencing. Genome Res 20, 265–272 (2010). 20019144 10.1101/gr.097261.109 PMC2813482

[b18] Liu C., Finegold S. M., Song Y. & Lawson P. A. Reclassification of *Clostridium* coccoides, *Ruminococcus* hansenii, *Ruminococcus* hydrogenotrophicus, *Ruminococcus* luti, *Ruminococcus* productus and *Ruminococcus* schinkii as Blautia coccoides gen. nov., comb. nov., Blautia hansenii comb. nov., Blautia hydrogenotrophica comb. nov., Blautia luti comb. nov., Blautia producta comb. nov., Blautia schinkii comb. nov. and description of Blautia wexlerae sp. nov., isolated from human faeces. Int J Syst Evol Microbiol 58, 1896–1902 (2008). 18676476 10.1099/ijs.0.65208-0

[b19] Benus R. F. *et al.* Association between *Faecalibacterium prausnitzii* and dietary fibre in colonic fermentation in healthy human subjects. Br J Nutr 104, 693–700 (2010). 20346190 10.1017/S0007114510001030

[b20] Hespell R. B., Wolf R. & Bothast R. J. Fermentation of xylans by Butyrivibrio *fibrisolvens* and other ruminal bacteria. Appl Environ Microbiol 53, 2849–2853 (1987). 3124741 10.1128/aem.53.12.2849-2853.1987 PMC204211

[b21] Cantarel B. L. *et al.* The Carbohydrate-Active EnZymes database (CAZy): an expert resource for Glycogenomics. Nucleic Acids Res 37, D233–D238 (2009). 18838391 10.1093/nar/gkn663 PMC2686590

[b22] Kanehisa M., Goto S., Kawashima S., Okuno Y. & Hattori M. The KEGG resource for deciphering the genome. Nucleic Acids Res 32, D277–D280 (2004). 14681412 10.1093/nar/gkh063 PMC308797

[b23] Cantarel B. L., Lombard V. & Henrissat B. Complex carbohydrate utilization by the healthy human microbiome. PLoS One 7, e28742 (2012). 22719820 10.1371/journal.pone.0028742 PMC3374616

[b24] Miller D. A. *et al.* Complete genome sequence of the cellulose-degrading bacterium *Cellulosilyticum lentocellum*. J Bacteriol 193, 2357–2358 (2011). 21398547 10.1128/JB.00239-11 PMC3133088

[b25] Yin Y., Chen H., Hahn M. G., Mohnen D. & Xu Y. Evolution and function of the plant cell wall synthesis-related glycosyltransferase family 8. Plant Physiol 153, 1729–1746 (2010). 20522722 10.1104/pp.110.154229 PMC2923890

[b26] Joyce S. A. & Gahan C. G. The gut microbiota and the metabolic health of the host. Curr Opin Gastroenterol 30, 120–127 (2014). 24468803 10.1097/MOG.0000000000000039

[b27] Looft T. *et al.* In-feed antibiotic effects on the swine intestinal microbiome. Proc Natl Acad Sci USA 109, 1691–1696 (2012). 22307632 10.1073/pnas.1120238109 PMC3277147

[b28] Allen H. K. *et al.* Antibiotics in feed induce prophages in swine fecal microbiomes. MBio 2, 1867–1877 (2011). 10.1128/mBio.00260-11 PMC3225969 22128350

[b29] Isaacson R. & Kim H. B. The intestinal microbiome of the pig. Anim Health Res Rev 13, 100–109 (2012). 22853934 10.1017/S1466252312000084

[b30] Mao S., Zhang M., Liu J. & Zhu W. Characterising the bacterial microbiota across the gastrointestinal tracts of dairy cattle: membership and potential function. Sci Rep 5, 16116 (2015). 26527325 10.1038/srep16116 PMC4630781

[b31] Looft T. *et al.* Bacteria, phages and pigs: the effects of in-feed antibiotics on the microbiome at different gut locations. ISME J 8, 1566–1576 (2014). 24522263 10.1038/ismej.2014.12 PMC4817603

[b32] Zoetendal E. G. *et al.* The human small intestinal microbiota is driven by rapid uptake and conversion of simple carbohydrates. ISME J 6, 1415–1426 (2012). 22258098 10.1038/ismej.2011.212 PMC3379644

[b33] Zhao L. The gut microbiota and obesity: from correlation to causality. Nat Rev Microbiol 11, 639–647 (2013). 23912213 10.1038/nrmicro3089

[b34] Million M., Lagier J. C., Yahav D. & Paul M. Gut bacterial microbiota and obesity. Clin Microbiol Infect 19, 305–313 (2013). 23452229 10.1111/1469-0691.12172

[b35] Angelakis E., Armougom F., Million M. & Raoult D. The relationship between gut microbiota and weight gain in humans. Future Microbiol 7, 91–109 (2012). 22191449 10.2217/fmb.11.142

[b36] Cani P. D. *et al.* Metabolic endotoxemia initiates obesity and insulin resistance. Diabetes 56, 1761–1772 (2007). 17456850 10.2337/db06-1491

[b37] Khanna S. & Pardi D. S. IBD: Poor outcomes after *Clostridium difficile* infection in IBD. Nat Rev Gastroenterol Hepatol 9, 307–308 (2012). 22547310 10.1038/nrgastro.2012.87

[b38] Ding S. *et al.* High-fat diet: bacteria interactions promote intestinal inflammation which precedes and correlates with obesity and insulin resistance in mouse. PLoS One 5, e12191 (2010). 20808947 10.1371/journal.pone.0012191 PMC2922379

[b39] Hildebrandt M. A. *et al.* High-fat diet determines the composition of the murine gut microbiome independently of obesity. Gastroenterology 137, 1–2 (2009). 10.1053/j.gastro.2009.08.042 PMC2770164 19706296

[b40] Wellen K. E. & Hotamisligil G. S. Obesity-induced inflammatory changes in adipose tissue. J Clin Invest 112, 1785–1788 (2003). 14679172 10.1172/JCI20514 PMC297006

[b41] Verdam F. J. *et al.* Human intestinal microbiota composition is associated with local and systemic inflammation in obesity. Obesity 21, E607–E615 (2013). 23526699 10.1002/oby.20466

[b42] Ivarsson E., Roos S., Liu H. Y. & Lindberg J. E. Fermentable non-starch polysaccharides increases the abundance of *Bacteroides*-*Prevotella*-*Porphyromonas* in ileal microbial community of growing pigs. Animal 8, 1777–1787 (2014). 25046106 10.1017/S1751731114001827

[b43] Ridaura V. K. *et al.* Gut microbiota from twins discordant for obesity modulate metabolism in mice. Science 341, 1241214 (2013). 24009397 10.1126/science.1241214 PMC3829625

[b44] Neyrinck A. M. *et al.* Prebiotic effects of wheat arabinoxylan related to the increase in bifidobacteria, Roseburia and *Bacteroides*/*Prevotella* in diet-induced obese mice. PLoS One 6, e20944 (2011). 21695273 10.1371/journal.pone.0020944 PMC3111466

[b45] Turnbaugh P. J. *et al.* A core gut microbiome in obese and lean twins. Nature 457, 480–484 (2009). 19043404 10.1038/nature07540 PMC2677729

[b46] Alm E., Huang K. & Arkin A. The evolution of two-component systems in bacteria reveals different strategies for niche adaptation. PLoS Comput Biol 2, e143 (2006). 17083272 10.1371/journal.pcbi.0020143 PMC1630713

[b47] Cui Y., Tu R., Wu L., Hong Y. & Chen S. A hybrid two-component system protein from Azospirillum brasilense Sp7 was involved in chemotaxis. Microbiol Res 166, 458–467 (2011). 20869221 10.1016/j.micres.2010.08.006

[b48] Sonnenburg E. D. *et al.* A hybrid two-component system protein of a prominent human gut symbiont couples glycan sensing *in vivo* to carbohydrate metabolism. Proc Natl Acad Sci USA 103, 8834–8839 (2006). 16735464 10.1073/pnas.0603249103 PMC1472243

[b49] Magoc T. & Salzberg S. L. FLASH: fast length adjustment of short reads to improve genome assemblies. Bioinformatics 27, 2957–2963 (2011). 21903629 10.1093/bioinformatics/btr507 PMC3198573

[b50] Majaneva M., Hyytiainen K., Varvio S. L., Nagai S. & Blomster J. Bioinformatic Amplicon Read Processing Strategies Strongly Affect Eukaryotic Diversity and the Taxonomic Composition of Communities. PLoS One 10, e0130035 (2015). 26047335 10.1371/journal.pone.0130035 PMC4457843

[b51] Wang Q., Garrity G. M., Tiedje J. M. & Cole J. R. Naive Bayesian classifier for rapid assignment of rRNA sequences into the new bacterial taxonomy. Appl Environ Microbiol 73, 5261–5267 (2007). 17586664 10.1128/AEM.00062-07 PMC1950982

[b52] Werner J. J., Zhou D., Caporaso J. G., Knight R. & Angenent L. T. Comparison of Illumina paired-end and single-direction sequencing for microbial 16S rRNA gene amplicon surveys. ISME J 6, 1273–1276 (2012). 22170427 10.1038/ismej.2011.186 PMC3379627

[b53] Parks D. H., Tyson G. W., Hugenholtz P. & Beiko R. G. STAMP: statistical analysis of taxonomic and functional profiles. Bioinformatics 30, 3123–3124 (2014). 25061070 10.1093/bioinformatics/btu494 PMC4609014

[b54] White J. R., Nagarajan N. & Pop M. Statistical methods for detecting differentially abundant features in clinical metagenomic samples. PLoS Comput Biol 5, e1000352 (2009). 19360128 10.1371/journal.pcbi.1000352 PMC2661018

[b55] Benjamini Y. & Hochberg Y. Controlling the false discovery rate: a practical and powerful approach to multiple testing. J R Stat Soc B 57, 289–300 (1995).

[b56] Qin J. *et al.* A metagenome-wide association study of gut microbiota in type 2 diabetes. Nature 490, 55–60 (2012). 23023125 10.1038/nature11450

[b57] Zhu W., Lomsadze A. & Borodovsky M. Ab initio gene identification in metagenomic sequences. Nucleic Acids Res 38, e132 (2010). 20403810 10.1093/nar/gkq275 PMC2896542

[b58] Li W. & Godzik A. Cd-hit: a fast program for clustering and comparing large sets of protein or nucleotide sequences. Bioinformatics 22, 1658–1659 (2006). 16731699 10.1093/bioinformatics/btl158

[b59] Li R. *et al.* SOAP2: an improved ultrafast tool for short read alignment. Bioinformatics 25, 1966–1967 (2009). 19497933 10.1093/bioinformatics/btp336

[b60] Storey J. D., Taylor J. E. & Siegmund D. Strong control, conservative point estimation and simultaneous conservative consistency of false discovery rates: a unified approach. J R Stat Soc B 66, 187–205 (2004).

