# Supplementary material for: Uncovering the composition of microbial community structure and metagenomics among three gut locations in pigs with distinct fatness
Source: Sci Rep. 2016 Jun 3;6:27427. doi: 10.1038/srep27427 (PMC4891666; doi:10.1038/srep27427)
Supplement: Supplementary Information [file srep27427-s1.doc]

# Uncovering the composition of microbial community structure and metagenomics among three gut locations in pigs with distinct fatness

Hui Yang^1^, Xiaochang Huang^1^, Shaoming Fang, Wenshui Xin, Lusheng Huang^*^, Congying Chen^*^

^1^ These authors contributed equally to this work

^*^ To whom correspondence should be addressed

*State Key Laboratory for Pig Genetic Improvement and Production Technology, Jiangxi Agricultural University, 330045, Nanchang, China*

**Address for correspondence**

Congying Chen, Lusheng Huang

State Key Laboratory of Pig Genetic Improvement and Production Technology Jiangxi Agricultural University

Nanchang, 330045, P. R. China

Phone: 0086-791-83813080

Fax: 0086-791-83900189

**Supplementary Figure S1: Relative abundance plots for samples from the three gut locations of distinct fatness pigs,** **summarized at the genus level from 16S rRNA sequencing data.** Samples are represented along the horizontal axis, and relative abundance is denoted by the vertical axis.


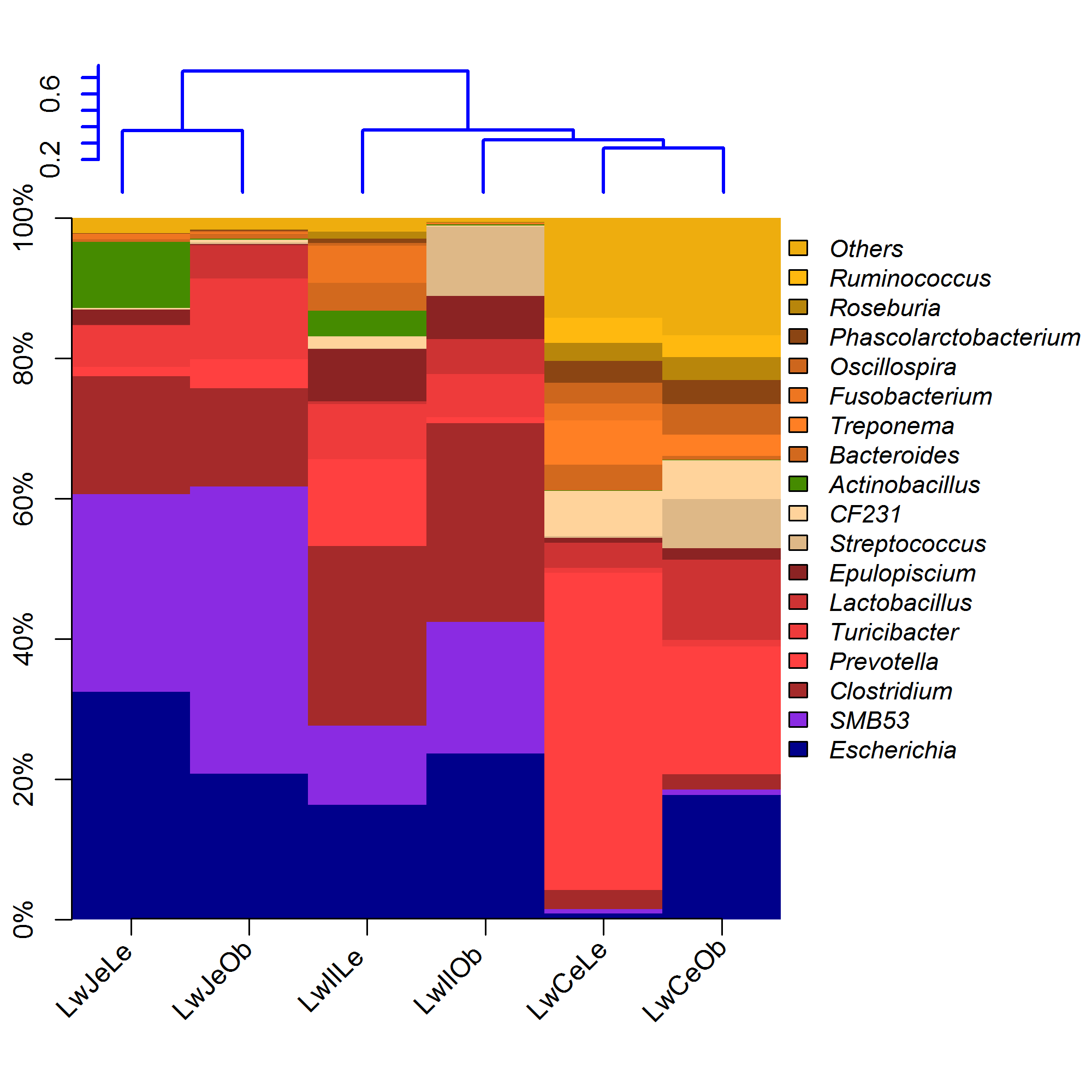


**Supplementary Figure S2: Comparisons of taxonomic profile of the microbiomes from the jejunum, ileum and cecum of high and low fatness pigs between 16S rRNA gene and metagenomic sequencing data at the** **bacterial phylum level.** Asterisks indicate the plots from metagenomic sequencing data.


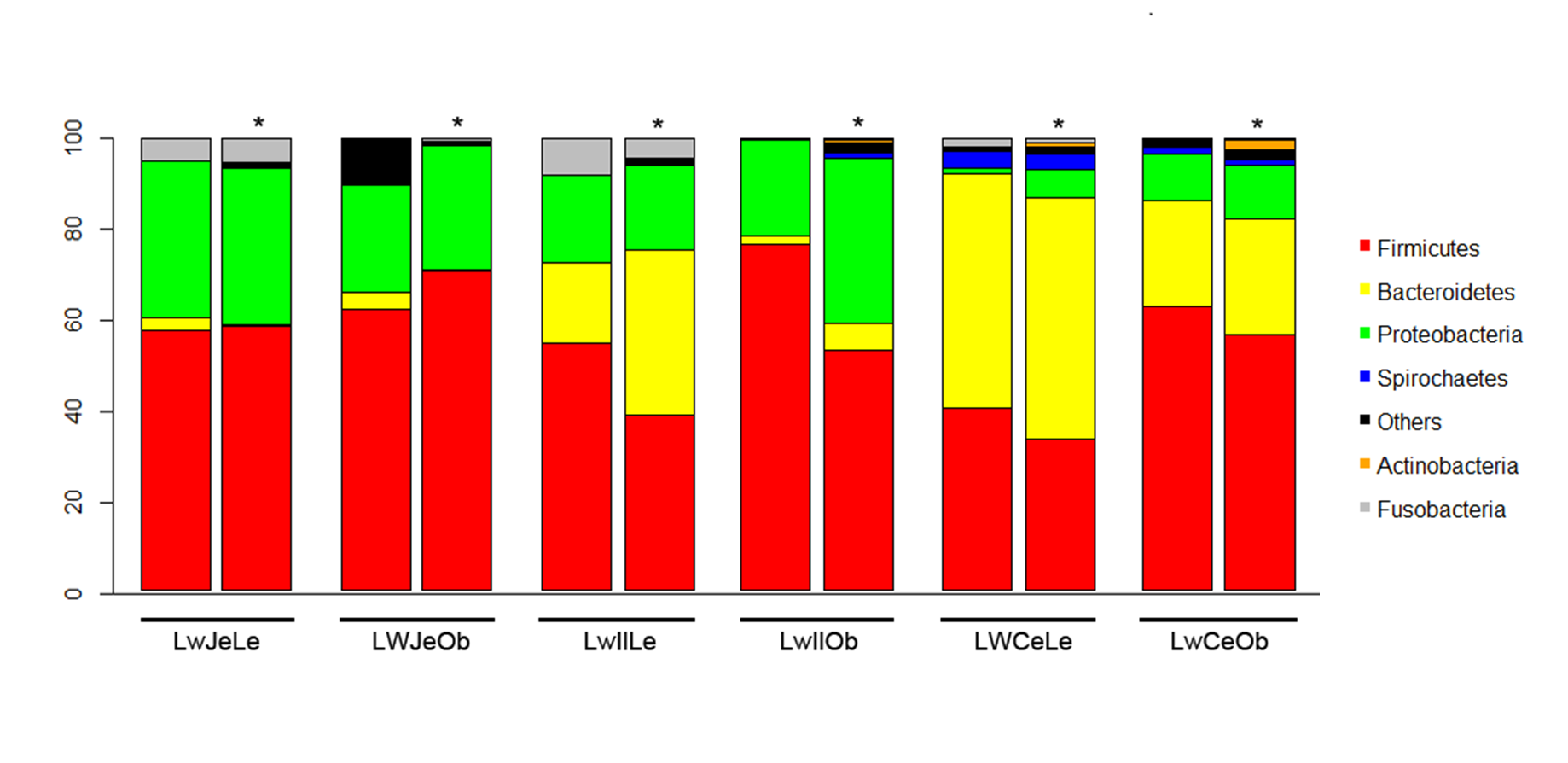


**Supplementary Figure S3:** **Principal component analysis revealed grouping of samples according to fatness.** Only the samples from the cecum were grouped following the fatness (C), suggesting the changes in bacterial diversity and composition.


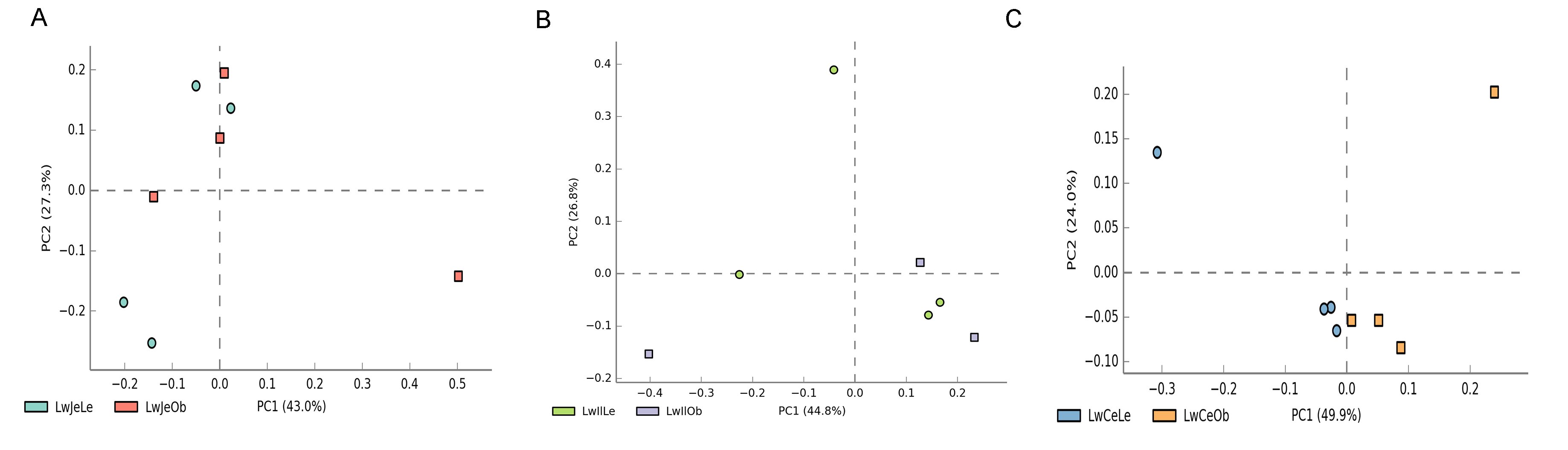


| **Taxa** | **Relative abundance** | | | **Kruskal-Wallis test** | | | ***Post-hoc* test** | | |
| --- | --- | --- | --- | --- | --- | --- | --- | --- | --- |
| **Genus** | **Jejunum**  **(Mean% ± SD)** | **Ileum**  **(Mean% ± SD)** | **Cecum**  **(Mean% ± SD)** | **P-value** | **Q-value** | **Effect size** | **Jejunum: Cecum**  **(P-value)** | **Ileum: Cecum**  **(P-value)** | **Jejunum: Ileum**  **(P-value)** |
| ***SMB53*** | 36.70 ± 19.54 | 14.54 ± 10.62 | 0.79 ± 0.831 | 5.58E-04 | 4.91E-03 | 0.57 | < 0.001 | < 0.01 | < 0.05 |
| ***Clostridium*** | 14.66 ± 13.52 | 27.14 ± 20.56 | 2.86 ± 3.77 | 1.33E-02 | 2.92E-02 | 0.32 | < 0.02 | < 0.01 | ≥ 0.1 |
| ***Bulleidia*** | 0.01 ± 0.02 | 0.02 ± 0.03 | 0.40 ± 0.39 | 2.89E-03 | 7.93E-03 | 0.38 | < 0.02 | < 0.05 | ≥ 0.1 |
| ***Rc4-4*** | 0.01 ± 0.02 | 0.07 ± 0.17 | 0.59 ± 0.58 | 8.14E-04 | 3.98E-03 | 0.34 | < 0.05 | < 0.05 | < 0.1 |
| ***Dorea*** | 0.01 ± 0.00 | 0.01 ± 0.01 | 0.60 ± 0.39 | 1.20E-03 | 4.42E-03 | 0.59 | < 0.001 | < 0.001 | ≥ 0.1 |
| ***YRC22*** | 0.03 ± 0.06 | 0.08 ± 0.18 | 0.62 ± 0.52 | 3.34E-03 | 8.16E-03 | 0.41 | < 0.02 | < 0.05 | ≥ 0.1 |
| ***02d06*** | 0.01 ± 0.03 | 0.01 ± 0.01 | 0.88 ± 0.77 | 3.65E-04 | 8.03E-03 | 0.45 | < 0.01 | < 0.01 | ≥ 0.1 |
| ***Blautia*** | 0.01 ± 0.01 | 0.02 ± 0.02 | 0.98 ± 0.77 | 4.15E-04 | 6.08E-03 | 0.51 | < 0.01 | < 0.01 | ≥ 0.1 |
| ***P-75-a5*** | 0.04 ± 0.08 | 0.09 ± 0.16 | 1.27 ± 0.97 | 6.08E-04 | 3.82E-03 | 0.50 | < 0.01 | < 0.01 | ≥ 0.1 |
| ***Faecalibacterium*** | 0.01 ± 0.01 | 0.02 ± 0.02 | 1.54 ± 1.91 | 9.18E-04 | 4.04E-03 | 0.29 | < 0.1 | < 0.1 | ≥ 0.1 |
| ***Coprococcus*** | 0.12 ± 0.11 | 0.29 ± 0.34 | 2.51 ± 1.61 | 1.45E-03 | 4.90E-03 | 0.57 | < 0.001 | < 0.01 | ≥ 0.1 |
| ***Roseburia*** | 0.03 ± 0.06 | 0.62 ± 1.35 | 3.00 ± 1.54 | 9.89E-04 | 3.96E-03 | 0.55 | < 0.001 | < 0.01 | ≥ 0.1 |
| ***Phascolarctobacterium*** | 0.10 ± 0.20 | 0.33 ± 0.79 | 3.21 ± 0.84 | 5.51E-04 | 6.06E-03 | 0.82 | < 0.001 | < 0.001 | ≥ 0.1 |
| ***Ruminococcus*** | 0.02 ± 0.03 | 0.07 ± 0.13 | 3.64 ± 2.68 | 5.88E-04 | 4.31E-03 | 0.54 | < 0.001 | < 0.01 | ≥ 0.1 |
| ***Oscillospira*** | 0.12 ± 0.25 | 0.23 ± 0.51 | 3.76 ± 2.21 | 6.86E-04 | 3.77E-03 | 0.61 | < 0.001 | < 0.001 | ≥ 0.1 |
| ***Treponema*** | 0.03 ± 0.03 | 0.07 ± 0.14 | 5.49 ± 6.51 | 2.32E-03 | 6.81E-03 | 0.31 | < 0.05 | < 0.05 | ≥ 0.1 |
| ***CF231*** | 0.30 ± 0.51 | 1.01 ± 2.27 | 6.39 ± 4.87 | 1.81E-03 | 5.69E-03 | 0.44 | < 0.01 | < 0.02 | ≥ 0.1 |
| ***Prevotella*** | 2.72 ± 5.26 | 7.13 ± 16.22 | 31.35 ± 15.46 | 3.25E-03 | 8.41E-03 | 0.49 | < 0.01 | < 0.01 | ≥ 0.1 |

**Supplementary Table S1. Microbial genera that had different enrichment among three gut locations based on 16S rRNA gene-sequencing data with** **Kruskal-Wallis test.**

**Supplementary Table S2. Bacterial species that were significantly enriched in each gut location based on metagenomic sequencing data.**

| **Taxa** | **Relative abundance** | | | **ANOVA test** | | | **Post-hoc test** | | |
| --- | --- | --- | --- | --- | --- | --- | --- | --- | --- |
| **Species** | **Jejunum**  **(Mean% ± SD)** | **Ileum**  **(Mean% ± SD)** | **Cecum**  **(Mean% ± SD)** | **P-value** | **Q-value** | **Effect size** | **Jejunum: Cecum (P-value)** | **Ileum: Cecum (P-value)** | **Jejunum: Ileum**  **(P-value)** |
| ***Clostridium acetobutylicum*** | 0.44 ± 0.03 | 0.30 ± 0.06 | 0.08 ± 0.02 | 1.85E-02 | 1.52E-01 | 0.93 | < 0.02 | < 0.1 | ≥ 0.1 |
| ***Clostridium acidurici*** | 0.35 ± 0.02 | 0.25 ± 0.02 | 0.07 ± 0.03 | 7.70E-03 | 1.14E-01 | 0.96 | < 0.01 | < 0.05 | ≥ 0.1 |
| ***Clostridium beijerinckii*** | 4.05 ± 0.44 | 2.18 ± 0.22 | 0.28 ± 0.07 | 6.19E-03 | 1.15E-01 | 0.97 | < 0.01 | < 0.05 | < 0.05 |
| ***Clostridium botulinum*** | 10.53 ± 0.60 | 6.52 ± 1.01 | 1.00 ± 0.27 | 5.37E-03 | 1.14E-01 | 0.97 | < 0.01 | < 0.05 | < 0.1 |
| ***Clostridium butyricum*** | 0.18 ± 0.01 | 0.09 ± 0.02 | 0.01 ± 0.00 | 1.06E-02 | 1.05E-01 | 0.95 | < 0.01 | < 0.1 | < 0.1 |
| ***Clostridium cellulovorans*** | 1.12 ± 0.05 | 0.76 ± 0.13 | 0.21 ± 0.06 | 1.20E-02 | 1.11E-01 | 0.95 | < 0.02 | < 0.05 | ≥ 0.1 |
| ***Clostridium ljungdahlii*** | 0.53 ± 0.03 | 0.36 ± 0.03 | 0.08 ± 0.03 | 4.00E-03 | 1.18E-01 | 0.97 | < 0.01 | < 0.02 | < 0.1 |
| ***Clostridium novyi*** | 0.56 ± 0.01 | 0.35 ± 0.07 | 0.08 ± 0.02 | 9.29E-03 | 1.25E-01 | 0.96 | < 0.01 | < 0.05 | < 0.1 |
| ***Clostridium pasteurianum*** | 0.50 ± 0.00 | 0.39 ± 0.08 | 0.08 ± 0.04 | 2.14E-02 | 1.67E-01 | 0.92 | < 0.05 | < 0.05 | ≥ 0.1 |
| ***Clostridium perfringens*** | 6.95 ± 0.31 | 5.70 ± 0.48 | 0.64 ± 0.16 | 1.91E-03 | 1.41E-01 | 0.98 | < 0.01 | < 0.01 | ≥ 0.1 |
| ***Clostridium saccharoperbutylacetonicum*** | 5.25 ± 0.54 | 2.87 ± 0.39 | 0.40 ± 0.10 | 7.13E-03 | 1.17E-01 | 0.96 | < 0.01 | < 0.05 | < 0.05 |
| ***Clostridium tetani*** | 0.43 ± 0.00 | 0.27 ± 0.03 | 0.07 ± 0.02 | 2.46E-03 | 1.21E-01 | 0.98 | < 0.01 | < 0.02 | < 0.05 |
| ***Enterobacter cloacae*** | 0.12 ± 0.01 | 0.07 ± 0.01 | 0.03 ± 0.01 | 9.42E-03 | 1.16E-01 | 0.96 | < 0.01 | < 0.1 | < 0.1 |
| ***Acidaminococcus intestini*** | 0.00 ± 0.00 | 0.04 ± 0.03 | 0.20 ± 0.02 | 9.75E-03 | 1.11E-01 | 0.95 | < 0.01 | < 0.02 | ≥ 0.1 |
| ***Butyrivibrio fibrisolvens*** | 0.09 ± 0.04 | 0.09 ± 0.01 | 0.71 ± 0.19 | 4.81E-02 | 2.85E-01 | 0.87 | < 0.1 | < 0.1 | ≥ 0.1 |
| ***Butyrivibrio proteoclasticus*** | 0.07 ± 0.03 | 0.08 ± 0.03 | 1.20 ± 0.23 | 1.51E-02 | 1.31E-01 | 0.94 | < 0.02 | < 0.05 | ≥ 0.1 |
| ***Clostridium cellulosi*** | 0.01 ± 0.01 | 0.01 ± 0.00 | 0.15 ± 0.03 | 2.27E-02 | 1.68E-01 | 0.92 | < 0.05 | < 0.05 | ≥ 0.1 |
| ***Eubacterium siraeum*** | 0.04 ± 0.02 | 0.05 ± 0.03 | 1.03 ± 0.13 | 4.01E-03 | 9.88E-02 | 0.97 | < 0.01 | < 0.01 | ≥ 0.1 |
| ***Faecalibacterium prausnitzii*** | 0.01 ± 0.00 | 0.16 ± 0.09 | 4.73 ± 0.07 | 2.52E-05 | 3.74E-03 | 1.00 | < 0.001 | < 0.001 | ≥ 0.1 |
| ***Ruminococcus albus*** | 0.00 ± 0.00 | 0.02 ± 0.01 | 0.54 ± 0.06 | 2.58E-03 | 9.53E-02 | 0.98 | < 0.01 | < 0.01 | ≥ 0.1 |
| ***Ruminococcus bromii*** | 0.02 ± 0.00 | 0.03 ± 0.02 | 1.09 ± 0.19 | 9.92E-03 | 1.05E-01 | 0.95 | < 0.02 | < 0.02 | ≥ 0.1 |

**Supplementary Table S3. CAZy functional terms that showed different enrichment in each gut location.**

| **CAZY** | **Relative abundance** | | | **ANOVA test** | | | ***Post-hoc* test** | | |
| --- | --- | --- | --- | --- | --- | --- | --- | --- | --- |
| **Subclass 2** | **Jejunum**  **(Mean% ± SD)** | **Ileum**  **(Mean% ± SD)** | **Cecum**  **(Mean% ± SD)** | **P-value** | **Q-value** | **Effect size** | **Jejunum: Cecum (P-value)** | **Ileum: Cecum**  **(P-value)** | **Jejunum: Ileum (P-value)** |
| GT25 | 0.30 ± 0.05 | 0.07 ± 0.03 | 0.02 ± 0.01 | 2.22E-02 | 3.17E-02 | 0.92 | < 0.05 | < 0.05 | ≥ 0.1 |
| GT26 | 2.01 ± 0.19 | 1.75 ± 0.14 | 1.04 ± 0.09 | 4.09E-02 | 4.96E-02 | 0.88 | < 0.05 | < 0.1 | ≥ 0.1 |
| CBM12 | 0.49 ± 0.07 | 0.25 ± 0.06 | 0.04 ± 0.00 | 1.99E-02 | 3.17E-02 | 0.93 | < 0.02 | < 0.1 | ≥ 0.1 |
| GH28 | 0.08 ± 0.00 | 0.53 ± 0.08 | 1.67 ± 0.00 | 3.34E-04 | 6.68E-03 | 1.00 | < 0.01 | < 0.01 | < 0.02 |
| GH51 | 0.67 ± 0.16 | 1.32 ± 0.04 | 2.66 ± 0.06 | 1.88E-03 | 1.62E-02 | 0.98 | < 0.01 | < 0.01 | < 0.05 |
| GH43 | 0.99 ± 0.34 | 2.88 ± 0.43 | 7.00 ± 0.10 | 2.03E-03 | 1.62E-02 | 0.98 | < 0.01 | < 0.01 | < 0.1 |
| CBM35 | 0.13 ± 0.05 | 0.58 ± 0.01 | 1.98 ± 0.05 | 1.25E-04 | 5.01E-03 | 1.00 | < 0.001 | < 0.001 | < 0.01 |
| CE6 | 0.43 ± 0.12 | 2.08 ± 0.18 | 6.08 ± 0.81 | 8.08E-03 | 3.08E-02 | 0.96 | < 0.01 | < 0.05 | ≥ 0.1 |
| CE7 | 0.72 ± 0.43 | 0.81 ± 0.20 | 3.12 ± 0.31 | 2.32E-02 | 3.20E-02 | 0.92 | < 0.05 | < 0.05 | ≥ 0.1 |
| CE12 | 0.06 ± 0.06 | 1.15 ± 0.48 | 6.10 ± 1.12 | 1.73E-02 | 3.11E-02 | 0.93 | < 0.02 | < 0.05 | ≥ 0.1 |
| CE15 | 0.01 ± 0.01 | 0.50 ± 0.14 | 1.61 ± 0.29 | 2.14E-02 | 3.17E-02 | 0.92 | < 0.05 | < 0.1 | ≥ 0.1 |
| GH2 | 6.79 ± 0.66 | 9.47 ± 0.53 | 10.84 ± 0.01 | 2.14E-02 | 3.17E-02 | 0.92 | < 0.02 | ≥ 0.1 | < 0.1 |
| GH5 | 0.22 ± 0.05 | 0.69 ± 0.16 | 1.87 ± 0.08 | 3.88E-03 | 2.58E-02 | 0.98 | < 0.01 | < 0.01 | ≥ 0.1 |
| GH10 | 0.01 ± 0.01 | 0.16 ± 0.04 | 0.56 ± 0.06 | 6.42E-03 | 3.08E-02 | 0.97 | < 0.01 | < 0.02 | ≥ 0.1 |
| GH27 | 0.04 ± 0.02 | 0.18 ± 0.06 | 0.52 ± 0.00 | 7.16E-03 | 3.08E-02 | 0.96 | < 0.01 | < 0.02 | ≥ 0.1 |
| GH30 | 0.04 ± 0.01 | 0.19 ± 0.08 | 0.61 ± 0.06 | 1.34E-02 | 3.08E-02 | 0.94 | < 0.02 | < 0.05 | ≥ 0.1 |
| GH44 | 0.00 ± 0.00 | 0.00 ± 0.00 | 0.01 ± 0.00 | 3.18E-02 | 4.08E-02 | 0.90 | < 0.05 | < 0.05 | ≥ 0.1 |
| GH66 | 0.00 ± 0.00 | 0.05 ± 0.02 | 0.14 ± 0.01 | 1.75E-02 | 3.11E-02 | 0.93 | < 0.02 | < 0.05 | ≥ 0.1 |
| GH78 | 0.03 ± 0.03 | 0.26 ± 0.08 | 1.19 ± 0.19 | 1.29E-02 | 3.08E-02 | 0.94 | < 0.02 | < 0.05 | ≥ 0.1 |
| GH79 | 0.00 ± 0.00 | 0.01 ± 0.01 | 0.05 ± 0.00 | 3.26E-02 | 4.08E-02 | 0.90 | < 0.05 | < 0.1 | ≥ 0.1 |
| GH105 | 0.20 ± 0.12 | 0.43 ± 0.09 | 1.08 ± 0.01 | 1.22E-02 | 3.08E-02 | 0.95 | < 0.02 | < 0.05 | ≥ 0.1 |
| GH106 | 0.00 ± 0.00 | 0.16 ± 0.02 | 0.54 ± 0.08 | 7.92E-03 | 3.08E-02 | 0.96 | < 0.01 | < 0.02 | ≥ 0.1 |
| GH115 | 0.09 ± 0.03 | 0.40 ± 0.12 | 1.06 ± 0.09 | 1.04E-02 | 3.08E-02 | 0.95 | < 0.01 | < 0.05 | ≥ 0.1 |
| GT10 | 0.00 ± 0.00 | 0.11 ± 0.07 | 0.38 ± 0.00 | 1.71E-02 | 3.11E-02 | 0.93 | < 0.02 | < 0.05 | ≥ 0.1 |
| GT11 | 0.07 ± 0.02 | 0.11 ± 0.03 | 0.29 ± 0.03 | 2.18E-02 | 3.17E-02 | 0.92 | < 0.05 | < 0.05 | ≥ 0.1 |
| CBM4 | 0.12 ± 0.08 | 0.09 ± 0.00 | 0.59 ± 0.05 | 1.12E-02 | 3.08E-02 | 0.95 | < 0.02 | < 0.02 | ≥ 0.1 |
| CBM6 | 1.19 ± 0.31 | 2.12 ± 0.72 | 6.61 ± 0.53 | 1.15E-02 | 3.08E-02 | 0.95 | < 0.02 | < 0.05 | ≥ 0.1 |
| CBM13 | 0.11 ± 0.09 | 0.65 ± 0.04 | 1.95 ± 0.34 | 1.68E-02 | 3.11E-02 | 0.93 | < 0.02 | < 0.05 | ≥ 0.1 |
| CBM61 | 0.01 ± 0.01 | 0.07 ± 0.05 | 0.74 ± 0.13 | 1.39E-02 | 3.08E-02 | 0.94 | < 0.02 | < 0.05 | ≥ 0.1 |

**Supplementary Table S4. KEGG subsystems that showed significantly different enrichment in each gut location.**

| **Kegg** | **Relative abundance** | | | **ANOVA test** | | | **Post-hoc test** | | |
| --- | --- | --- | --- | --- | --- | --- | --- | --- | --- |
| **LEVEL 3** | **Jejunum**  **(Mean% ± SD)** | **Ileum**  **(Mean% ± SD)** | **Cecum**  **(Mean% ± SD)** | **P-value** | **Q-value** | **Effect size** | **Jejunum: Cecum (P-value)** | **Ileum: Cecum (P-value)** | **Jejunum: Ileum (P-value)** |
| **Phosphotransferase system (PTS)** | 0.79 ± 0.09 | 0.60 ± 0.01 | 0.26 ± 0.10 | 3.85E-02 | 8.80E-02 | 0.89 | < 0.05 | < 0.1 | ≥ 0.1 |
| **Phosphatidylinositol signaling system** | 0.17 ± 0.01 | 0.14 ± 0.03 | 0.04 ± 0.01 | 4.35E-02 | 8.80E-02 | 0.88 | < 0.05 | < 0.1 | ≥ 0.1 |
| **Butanoate metabolism** | 0.90 ± 0.02 | 0.81 ± 0.02 | 0.56 ± 0.08 | 4.29E-02 | 8.80E-02 | 0.88 | < 0.05 | < 0.1 | ≥ 0.1 |
| **Glycerolipid metabolism** | 0.54 ± 0.06 | 0.44 ± 0.01 | 0.29 ± 0.02 | 4.14E-02 | 8.80E-02 | 0.88 | < 0.05 | ≥ 0.1 | ≥ 0.1 |
| **Fatty acid metabolism** | 0.47 ± 0.03 | 0.41 ± 0.00 | 0.22 ± 0.03 | 9.87E-03 | 7.58E-02 | 0.95 | < 0.01 | < 0.05 | ≥ 0.1 |
| **Drug metabolism - cytochrome P450** | 0.46 ± 0.05 | 0.37 ± 0.04 | 0.15 ± 0.05 | 4.76E-02 | 8.93E-02 | 0.87 | < 0.05 | < 0.1 | ≥ 0.1 |
| **Tetracycline biosynthesis** | 0.29 ± 0.02 | 0.24 ± 0.00 | 0.11 ± 0.02 | 1.32E-02 | 7.67E-02 | 0.94 | < 0.02 | < 0.05 | ≥ 0.1 |
| **Stilbenoid, diarylheptanoid and gingerol biosynthesis** | 0.05 ± 0.00 | 0.07 ± 0.00 | 0.02 ± 0.00 | 1.01E-02 | 7.58E-02 | 0.95 | < 0.01 | < 0.01 | ≥ 0.1 |
| **Progesterone-mediated oocyte maturation** | 0.38 ± 0.02 | 0.48 ± 0.04 | 0.55 ± 0.00 | 4.08E-02 | 8.80E-02 | 0.88 | < 0.05 | < 0.05 | ≥ 0.1 |
| **NOD-like receptor signaling pathway** | 0.38 ± 0.02 | 0.47 ± 0.03 | 0.53 ± 0.00 | 4.19E-02 | 8.80E-02 | 0.88 | < 0.05 | ≥ 0.1 | ≥ 0.1 |
| **Meiosis - yeast** | 0.05 ± 0.00 | 0.07 ± 0.00 | 0.16 ± 0.02 | 1.43E-02 | 7.67E-02 | 0.94 | < 0.02 | < 0.05 | ≥ 0.1 |
| **D-Alanine metabolism** | 0.37 ± 0.03 | 0.39 ± 0.02 | 0.52 ± 0.01 | 4.66E-02 | 8.93E-02 | 0.87 | < 0.05 | < 0.1 | ≥ 0.1 |
| **Taurine and hypotaurine metabolism** | 0.33 ± 0.01 | 0.33 ± 0.01 | 0.43 ± 0.00 | 6.96E-03 | 7.58E-02 | 0.96 | < 0.01 | < 0.01 | ≥ 0.1 |
| **Prenyltransferases** | 0.31 ± 0.00 | 0.34 ± 0.01 | 0.49 ± 0.03 | 2.53E-02 | 8.56E-02 | 0.91 | < 0.05 | < 0.05 | ≥ 0.1 |
| **Zeatin biosynthesis** | 0.26 ± 0.00 | 0.30 ± 0.03 | 0.49 ± 0.04 | 3.25E-02 | 8.80E-02 | 0.90 | < 0.05 | < 0.1 | ≥ 0.1 |
| **N-Glycan biosynthesis** | 0.09 ± 0.03 | 0.14 ± 0.07 | 0.41 ± 0.02 | 3.32E-02 | 8.80E-02 | 0.90 | < 0.05 | < 0.1 | ≥ 0.1 |
| **Phenylalanine, tyrosine and tryptophan biosynthesis** | 0.66 ± 0.02 | 0.68 ± 0.02 | 0.84 ± 0.03 | 1.79E-02 | 7.67E-02 | 0.93 | < 0.05 | < 0.05 | ≥ 0.1 |
| **Lipid biosynthesis proteins** | 0.59 ± 0.03 | 0.65 ± 0.01 | 0.89 ± 0.05 | 2.38E-02 | 8.56E-02 | 0.92 | < 0.05 | < 0.05 | ≥ 0.1 |
| **Cyanoamino acid metabolism** | 0.47 ± 0.06 | 0.47 ± 0.02 | 0.72 ± 0.00 | 2.57E-02 | 8.56E-02 | 0.91 | < 0.05 | < 0.05 | ≥ 0.1 |
| **Vitamin B6 metabolism** | 0.47 ± 0.00 | 0.49 ± 0.01 | 0.85 ± 0.02 | 5.17E-04 | 1.74E-02 | 0.99 | < 0.001 | < 0.001 | ≥ 0.1 |
| **Protein kinases** | 0.00 ± 0.00 | 0.00 ± 0.00 | 0.002 ± 0.00 | 2.25E-03 | 4.03E-02 | 0.98 | < 0.01 | < 0.01 | ≥ 0.1 |
| **Proximal tubule bicarbonate reclamation** | 0.00 ± 0.00 | 0.00 ± 0.00 | 0.06 ± 0.00 | 5.82E-04 | 1.74E-02 | 0.99 | < 0.001 | < 0.001 | ≥ 0.1 |

**Supplementary Table S5. Bacterial species that had distinctly relative abundance between high and low fatness pigs in the jejunum, ileum and cecum by metagenomic sequencing analysis.**

| **Location** | **Species** | **Low backfat (frequency %)** | **High backfat (frequency %)** | ***P*-values** | **Ratio of proportions** | **Difference between proportions** |
| --- | --- | --- | --- | --- | --- | --- |
| **Jejunum** | Actinobacillus succinogenes | 2.84 | 0.51 | 0.00 | 5.57 | 2.33 |
|  | Cellulosilyticum lentocellum | 6.12 | 1.72 | 0.00 | 3.56 | 4.40 |
|  | Fusobacterium nucleatum | 1.19 | 0.08 | 0.00 | 14.94 | 1.11 |
|  | Haemophilus influenzae | 1.32 | 0.25 | 0.00 | 5.30 | 1.07 |
|  | Mannheimia succiniciproducens | 1.50 | 0.27 | 0.00 | 5.56 | 1.23 |
|  | Clostridium phytofermentans | 0.38 | 2.38 | 0.00 | 6.23 | 2.00 |
|  | Escherichia fergusonii | 0.20 | 5.96 | 0.00 | 29.97 | 5.76 |
|  | Lactobacillus johnsonii | 0.02 | 7.85 | 0.00 | 364.92 | 7.82 |
| **Ileum** | Actinobacillus succinogenes | 1.41 | 0.00 | 0.00 | Invalid | 1.41 |
|  | Cellulosilyticum lentocellum | 2.06 | 0.68 | 0.00 | 3.01 | 1.38 |
|  | Bacteroides dorei | 1.81 | 0.12 | 0.00 | 15.17 | 1.69 |
|  | Bacteroides fragilis | 2.50 | 0.65 | 0.00 | 3.83 | 1.85 |
|  | Bacteroides helcogenes | 1.48 | 0.28 | 0.00 | 5.39 | 1.21 |
|  | Bacteroides salanitronis | 5.27 | 0.36 | 0.00 | 14.46 | 4.90 |
|  | Bacteroides thetaiotaomicron | 1.53 | 0.20 | 0.00 | 7.60 | 1.32 |
|  | Bacteroides vulgatus | 1.51 | 0.10 | 0.00 | 15.65 | 1.42 |
|  | Bacteroides xylanisolvens | 1.30 | 0.21 | 0.00 | 6.25 | 1.09 |
|  | Barnesiella viscericola | 1.72 | 0.25 | 0.00 | 7.02 | 1.48 |
|  | Prevotella dentalis | 5.34 | 1.00 | 0.00 | 5.32 | 4.33 |
|  | Prevotella denticola | 2.34 | 0.54 | 0.00 | 4.31 | 1.80 |
|  | Prevotella ruminicola | 4.62 | 1.03 | 0.00 | 4.47 | 3.59 |
|  | Chamaesiphon minutus | 0.00 | 1.27 | 0.00 | Invalid | 1.27 |
|  | Clostridium difficile | 6.07 | 16.02 | 0.00 | 2.64 | 9.95 |
|  | Clostridium sordellii | 5.68 | 15.29 | 0.00 | 2.69 | 9.61 |
|  | Escherichia coli | 4.64 | 16.96 | 0.00 | 3.65 | 12.32 |
|  | Sphaerochaeta coccoides | 0.01 | 1.36 | 0.00 | 178.44 | 1.35 |
|  | Vibrio harveyi | 0.00 | 2.40 | 0.00 | 941.90 | 2.39 |
| **Cecum** | Bacteroides dorei | 1.87 | 0.45 | 0.00 | 4.15 | 1.42 |
|  | Bacteroides fragilis | 3.46 | 1.47 | 0.00 | 2.35 | 1.98 |
|  | Bacteroides helcogenes | 2.32 | 1.06 | 0.00 | 2.20 | 1.27 |
|  | Bacteroides salanitronis | 5.29 | 1.53 | 0.00 | 3.45 | 3.75 |
|  | Bacteroides thetaiotaomicron | 1.93 | 0.76 | 0.00 | 2.56 | 1.18 |
|  | Megamonas hypermegale | 1.68 | 0.19 | 0.00 | 8.70 | 1.48 |
|  | Megasphaera elsdenii | 2.62 | 0.17 | 0.00 | 15.05 | 2.44 |
|  | Prevotella ruminicola | 8.96 | 4.18 | 0.00 | 2.14 | 4.78 |
|  | Treponema succinifaciens | 3.37 | 0.70 | 0.00 | 4.82 | 2.67 |
|  | Escherichia coli | 0.72 | 2.76 | 0.00 | 3.81 | 2.04 |
|  | Eubacterium rectale | 1.26 | 2.68 | 0.00 | 2.13 | 1.42 |
|  | Oscillibacter valericigenes | 1.38 | 6.62 | 0.00 | 4.82 | 5.25 |
|  | Parabacteroides distasonis | 0.85 | 1.93 | 0.00 | 2.28 | 1.08 |
|  | Roseburia hominis | 0.73 | 1.75 | 0.00 | 2.41 | 1.03 |
|  | Roseburia intestinalis | 1.24 | 3.22 | 0.00 | 2.61 | 1.99 |

**Species with a ratio of proportions < 2 and proportion difference < 1 between two samples were filtered**.

| **Supplementary Table S6. The Accession number of 16S rRNA survey and**  **metagenome data.**   | **Data Type** | **Sample Name** | **Accession NO** | | --- | --- | --- | | **16S rRNA data** | LwJeOb.1 | SRR3180648 | | LwJeOb.2 | SRR3180650 | | LwJeOb.3 | SRR3180653 | | LwJeOb.4 | SRR3180654 | | LwJeLe.5 | SRR3180655 | | LwJeLe.6 | SRR3180656 | | LwJeLe.7 | SRR3180657 | | LwJeLe.8 | SRR3180658 | | LwIlOb.2 | SRR3180666 | | LwIlOb.3 | SRR3334759 | | LwIlOb.4 | SRR3334760 | | LwIlLe.5 | SRR3334769 | | LwIlLe.6 | SRR3334767 | | LwIlLe.7 | SRR3334770 | | LwIlLe.8 | SRR3334778 | | LwCeOb.1 | SRR3334781 | | LwCeOb.2 | SRR3334785 | | LwCeOb.3 | SRR3334786 | | LwCeOb.4 | SRR3334807 | | LwCeLe.5 | SRR3334821 | | LwCeLe.6 | SRR3334853 | | LwCeLe.7 | SRR3334872 | | LwCeLe.8 | SRR3334882 | | **Metagenome data** | LwJeOb | SRR3169810 | | LwJeLe | SRR3173844 | | LwIlOb | SRR3176533 | | LwIlLe | SRR3176534 | | LwCeOb | SRR3178346 | | LwCeLe | SRR3178347 | |
| --- | --- | --- | --- | --- | --- | --- | --- | --- | --- | --- | --- | --- | --- | --- | --- | --- | --- | --- | --- | --- | --- | --- | --- | --- | --- | --- | --- | --- | --- | --- | --- | --- | --- | --- | --- | --- | --- | --- | --- | --- | --- | --- | --- | --- | --- | --- | --- | --- | --- | --- | --- | --- | --- | --- | --- | --- | --- | --- | --- | --- | --- | --- | --- |
